# The Phantom Menace: latest findings on effector biology in the rice blast fungus

**DOI:** 10.1007/s42994-023-00099-4

**Published:** 2023-03-27

**Authors:** Jessie Fernandez

**Affiliations:** Department of Microbiology and Cell Science at University of Florida-Institute of Food and Agricultural Science, Gainesville, FL 32611 USA

**Keywords:** Rice blast, Effector, Secretion, Computational analysis, Plant immunity, Climate change

## Abstract

*Magnaporthe oryzae* is a hemibiotrophic fungus responsible for the economically devastating and recalcitrant rice blast disease. However, the blast fungus is not only restricted to rice plants as it can also infect wheat, millet, and other crops. Despite previous outstanding discoveries aimed to understand and control the disease, the fungus remains one of the most important pathogens that threatens global food security. To cause disease, *M. oryzae* initiates morphological changes to attach, penetrate, and colonize rice cells, all while suppressing plant immune defenses that would otherwise hinder its proliferation. As such, *M. oryzae* actively secretes a battery of small proteins called “effectors” to manipulate host machinery. In this review, we summarize the latest findings in effector identification, expression, regulation, and functionality. We review the most studied effectors and their roles in pathogenesis. Additionally, we discern the current methodologies to structurally catalog effectors, and we highlight the importance of climate change and its impact on the future of rice blast disease.

## Introduction

Fungal plant pathogens represent a huge economic impact on crop production across the world. To establish successful infections, fungal pathogens form an intimate relationship with their host to gain access to nutrients and be able to grow and reproduce. Thereby, fungi have adapted to different lifestyles to aggressively colonize plant tissues and cause disease. For instance, some fungi kill and feed on dead cells (necrotrophs), while others feed on living host cells (biotrophs) (Fernandez and Orth 2018). However, there is a group that displays both feeding lifestyles during infection which are known as hemibiotrophs. Because these fungal pathogens damage host cells through this feeding lifestyle, they are at a constant battle to not be recognized by plant immune systems.

Plants have evolved two complex waves of defense networks against microbial attacks. As sessile organisms, plants utilize these defense waves to perceive and respond quickly to pathogen infections (Jones and Dangl 2006; Ngou et al. 2022). Plants initially can detect pathogen-associated molecular patterns (PAMPs) through surface receptors to subsequently activate PAMP-triggered immunity (PTI). PTI activation promotes the accumulation of reactive oxygen species (ROS), cell wall reinforcements, and the induction of MAP kinase signaling cascades that promote the expression of immunity genes (Jones and Dangl 2006). The second wave of defense involves the recognition of pathogen-secreted molecules, known as effectors, inside the host cell by intracellular receptors, which induces effector-triggered immunity (ETI) (Ngou et al. 2022). ETI promotes a cell-death response at the infection site, known as the hypersensitive response (HR), upon effector recognition by resistant host genes. Activating PTI or ETI boosts plant disease resistance and restricts pathogen proliferation at the infection site (Bryan et al. 2000; Dodds et al. 2006; Sohn et al. 2007; Zipfel 2008).

Rice blast disease remains one of the most recalcitrant diseases threatening global food security. This disease is caused by the ascomycete, filamentous fungus, *Magnaporthe oryzae* (syn. *Pyricularia oryzae*) and is directly responsible for the loss of more than 30% of the harvested rice yearly (Pennisi 2010). Blast fungus has an incredible adaptability to its environment given that *M. oryzae* isolates from different geographical regions show great diversity (Zhong et al. 2018). To ensure survival, the blast fungus undergoes different developmental changes to sense the plant surface, penetrate the plant cuticle, proliferate inside rice cells, and ultimately complete its disease cycle (Fernandez and Orth 2018; Wilson and Talbot 2009). *M. oryzae* starts infections with a conidium that attaches on the leaf surface and germinates, forming an appressorium at the tip of the germ tube (Ryder et al. 2022). The appressorium is a specialized penetration structure that assists the fungus in breaking the leaf cuticle to enter plant cells. Appressorial adhesion to the host cell is crucial in this stage and *M. oryzae* promotes this adhesion through mucilage production. This production is determined by spermine metabolism, as spermine is able to mediate ROS levels in the endoplasmic reticulum (ER) and ensure proper protein folding (Rocha et al. 2020). Following adhesion, the inside of the appressorium builds turgor pressure through the migration of lipids and solutes from the conidium into the appressorium (De Jong et al. 1997). Appressorium formation and penetration are dependent on proper cell cycle division and autophagy processes (Marroquin-Guzman et al. 2017; Osés-Ruiz et al. 2017; Saunders et al. 2010; Veneault-Fourrey et al. 2006). Both processes are regulated by target of rapamycin (TOR) pathway, which ensures adequate nutrients are available for appressorial development and maturation (Marroquin-Guzman et al. 2017; Osés-Ruiz et al. 2017). Once inside, the fungus develops an invasive hypha (IH) that will invaginate the plant membrane forming the extra-invasive hyphal membrane (EIHM) compartment to separate the pathogen from the host cytoplasm. This EIHM compartment is one of the hallmarks of the biotrophic lifestyle in *M. oryzae*, a place in which the fungus IH is surrounded by host-derived membranes (Giraldo et al. 2013; Jones et al. 2021; Khang et al. 2010; Sun et al. 2018). The tip of the primary and secondary IH contains a structure known as the biotrophic interface complex (BIC), which is another hallmark of the *M. oryzae* biotrophic lifestyle (Khang et al. 2010). BIC is a plant membrane-rich novel structure that is coupled to hyphal differentiation from filamentous to bulbous IH growth inside the host cells. Upon penetration, BIC appears adjacent to primary invasive hyphal tips, but later localizes subapically to bulbous IH, which is required for disease development. Before the *M. oryzae* IH moves to adjacent living cells, the EIHM integrity is compromised, displaying host vacuole shrinkage and subsequently rupture (Jones et al. 2021; Mochizuki et al. 2015; Sun et al. 2018). The IH will aggressively continue moving from cell to cell for a few days until the fungus switches to the necrotrophy lifestyle, killing the cell and developing necrotic lesions on rice leaves. This infection process is contingent on *M. oryzae*’s ability to sequester ROS levels that would otherwise trigger a host defense response (Li et al. 2020).

In addition to the tightly regulated developmental differentiation of *M.* oryzae, an important stipulation to establish successful infections involves *M. oryzae* actively secreting a repertoire of effectors into the host cell following appressorium maturation. The delivery of these effectors into plants is essential to interfere with PTI and ETI responses and promote disease progression. This review intends to highlight the recent findings on the biology of *M. oryzae* effectors and their molecular mechanisms controlling plant responses in a comprehensive manner. In addition, we will accentuate the effects of some environmental factors potentially influencing rice blast disease and emphasize the need for further characterization of climate change and rice blast disease studies.

## Ways to deliver effectors into rice cells

At what point during *M. oryzae* infection does effector secretion begin? It has been speculated that *M. oryzae* effector secretion starts from the base of the appressoria before IH formation. Recent studies demonstrate that the hemibiotrophic fungus, *Colletotrichum higginsianum,* focally secretes effectors into the cell from the appressorial penetration pores before IH formation (Kleemann et al. 2012). Moreover, it was found that the long-distance communication between the invading distal hyphal tip and nucleus is necessary to coordinate effector transcription and secretion (Bielska et al. 2014). Interestingly, long-distance retrograde motility of early endosomes was shown to be required to regulate the effector production during *Ustilago maydis* plant infections (Bielska et al. 2014). To this end, the blast fungus secretes a few effectors that could be inferred to be directly secreted from the base of the appressoria. For instance, the effector Bas170 was reported to accumulate at the host nuclei before primary IH formation and differentiation, and was found to localize in vesicles at the base of the appressoria (Oliveira-Garcia et al. 2022). This finding clearly suggests that the fungus utilizes some effectors that are secreted and translocated before direct pathogen invasion to prepare the terrain for the upcoming attack.

Many plant pathogens, especially those that prefer living cells, secrete a variety of molecules into host cells to evade recognition and rapidly conquer host tissues (Toruño et al. 2016). The mechanisms of effector delivery by some pathogens have been extensively studied. For instance, bacterial pathogens utilize a macromolecule machinery, needle-like apparatus referred to as type III secretion systems (T3SS) to inject effector proteins directly into host cells (Chang et al. 2014). The T3SS needle serves as a connector between the bacterial and host cells, allowing entry of bacterial virulent effectors into the host cytoplasm (Chang et al. 2014; Toruño et al. 2016). However, no delivery machinery equivalent to bacterial T3SS has been found in *M. oryzae* or any other fungal groups. The most comparable secretion machinery in fungi was recently found in *U. maydis,* where a heptameric protein complex, composed of five effector proteins and two membrane proteins, was shown to have similar functions in translocating effectors. Abolishment of the genes involved in this complex rendered *U. maydis* unable to suppress host defenses, suggesting an impairment in effector secretion (Ludwig et al. 2021). Regardless of the lack of delivery machinery, *M. oryzae* effectors have been shown to localize into the apoplastic space or host cytoplasmic sites (Giraldo et al. 2013). Apoplastic effectors (i.e., Slp1, Chia, and Ao1) are delivered and wildly dispersed into the extracellular space between the fungal cell wall and rice membrane acting as blockers of host detection (Giraldo et al. 2013; Khang et al. 2010; Mentlak et al. 2012). Cytoplasmic effectors (i.e., Bas1, HTRs, and AvrPiz-t), on the other hand, are secreted into the host cytoplasm through the BIC located at the primary and secondary IH (Fernandez and Orth 2018; Giraldo et al. 2013; Kim et al. 2020; Park et al. 2012). Some of the cytoplasmic effectors are able migrate to plant connections or healthy neighboring cells to prepare the host for the coming invasion.

Interestingly, *M. oryzae* effectors use two different secretion pathways in the cell (Giraldo et al. 2013). For instance, the conventional ER-Golgi secretion pathway plays an important role for the secretion of apoplastic effectors into the extracellular site of the plant (Giraldo et al. 2013). The secretion of these effectors is directly affected by treatments with Brefeldin A, an inhibitor of intracellular trafficking. By contrast, cytoplasmic effectors diverge from apoplastic effectors by using an unconventional pathway that involves the exocyst multisubunit complex and t-SNARE proteins to secrete and accumulate at the BIC site during biotrophic growth (Giraldo et al. 2013; Khang et al. 2010; Oliveira-Garcia et al. 2022). Following BIC secretion, recent findings suggest that the plant clathrin-mediated endocytosis process plays an important role in the internalization of *M. oryzae* cytoplasmic effectors in the host (Oliveira-Garcia et al. 2022). A combination of live-cell imaging, gene silencing, and inhibitor treatments unveiled that cytoplasmic effectors are localized at the BIC, which is enriched on plant membranes, actin and clathrin components, and packed into clathrin-coated vesicles (Oliveira-Garcia et al. 2022). However, the researchers argue that clathrin-independent endocytosis cannot be excluded from any involvement on the effector translocation. These findings provide new insights into the translocation of *M. oryzae* cytoplasmic effectors; however, there is still the need to understand the molecular mechanisms by which the fungus manipulates host cargo transport for its own benefit. Furthermore, it was recently shown that cytoplasmic effector secretion was dependent on tRNA modification and codon usage (Li et al. 2022). It was found that *M. oryzae* cytoplasmic effector mRNA translation in the host could only occur if proper tRNA thiolation modifications take place. Thus far, we are only beginning to decipher what factors determine effector regulation and destination in the invaded host cells or how *M. oryzae* effectors hijack the host machinery to travel across cells.

Most *M. oryzae* effectors do not share sequence similarities to known proteins and no obvious signal motifs in cell entry or targeting organelles have been identified. Despite no clear mark on their primary sequence, *M. oryzae* accurately secretes and highly expresses effectors only during plant infections and not in vegetative and developmental growth. How is the blast fungus able to timely regulate effector expression during pathogenicity? To address this intriguing question, a recent study demonstrated that a regulator of G-protein signaling, Rgs1, regulates the expression of at least 60 effector genes prior to *M. oryzae* biotrophic invasion (Tang et al. 2022). Rgs1 protein was previously shown to modulate G-protein subunits to allow *M. oryzae* to respond to physical cues during appressorium development (Liu et al. 2007; Yu et al. 2021). Interestingly, the N-terminal of the Rgs1 protein is the region responsible for independently regulating the transcription of *M. oryzae* effector genes (Tang et al. 2022). Moreover, Rgs1 overexpression led to fitness cost on *M. oryzae* pathogenicity, suggesting the importance of the repression of gene expression during developmental stages of the pathogen prior to infections. However, it is evident that the blast fungus secretes effectors into the host apoplastic site, and we wonder if this sophisticated mechanism regulated by Rgs1 also acts on apoplastic effectors or if it is localization specific. Further studies are needed to characterize and pinpoint additional key players involved in the timely regulated secretion mechanism of *M. oryzae* effectors.

## Most studied players in the arena

To this end, many *M. oryzae* effectors have been identified to be secreted during rice blast infections. However, only a handful of them have been functionally characterized to date (Böhnert et al. 2004; Mentlak et al. 2012; Park et al. 2012, 2016). This is mainly due to the functional redundancy effect among *M. oryzae* effectors and poor experimental approaches to accurately measure small changes in mutant phenotype, hindering the evaluation of precise effector contribution to the blast pathogenicity. However, the blast fungus generates an extensive number of effectors with intriguing subcellular localizations, and it can be suggested that either multiple effectors target the same host proteins, or one effector has several host targets. Ultimately, through effector secretions, the pathogen can puppeteer the host for its own benefit through suppressing host responses via controlling ROS accumulation and altering host transcription levels (Li et al. 2020). A comprehensive analysis of the currently identified fungal and oomycete effectors shows that 50% of effectors target mainly transcription and signaling pathways (He et al. 2020). The remaining half of effectors were identified to affect downstream functions such as general metabolism, cellular trafficking, RNA trafficking and processing, and protein regulation (He et al. 2020). It is evident that more advances are needed in studying the functionality of *M. oryzae* effectors to understand the infection dynamics in the broader context of how effectors function as a whole.

**Fig. 1:**
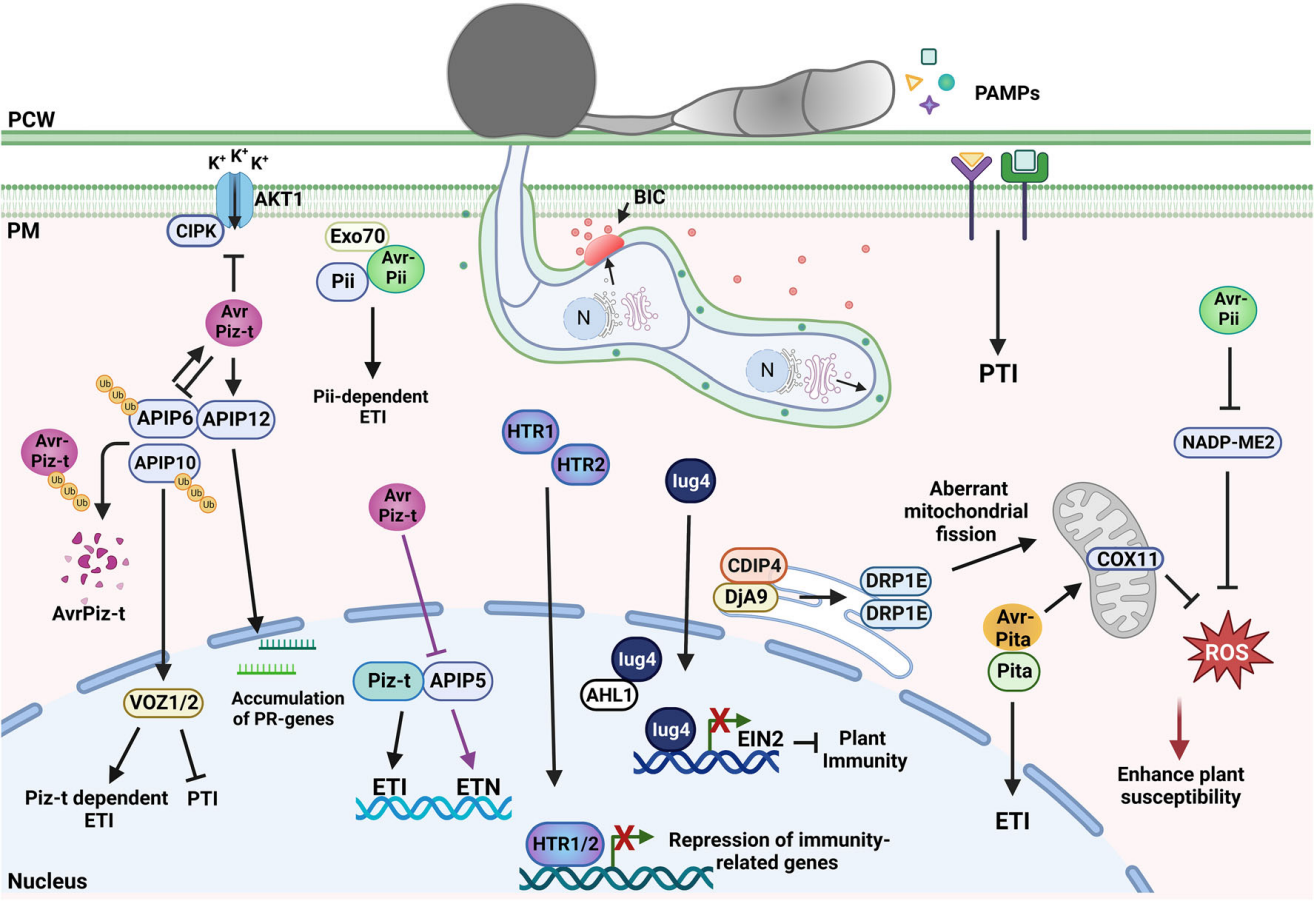
*M. oryzae* cytoplasmic effectors involved in modulating plant immune responses. Once *M. oryzae* attaches to the leaf cuticle and germinates, surface receptors are able to recognize PAMPs to induce PTI responses. To avoid recognition, *M. oryzae* secretes effectors such as AvrPiz-t, Avr-Pii, Avr-Pita, HTRs and Iug4, into the host cells to suppress rice immunity. The effector AvrPiz-t interacts with a variety of host targets to modulate defense responses. The interaction between AvrPiz-t and OsAPIP6 and OsAPIP10 causes AvrPiz-t degradation through ubiquitination that leads to regulation of PTI. In the absence of the cognate Piz-t, OsAPIP10 degradation promotes the accumulation of the transcription factors, OsVOZ1 and OsVOZ2, resulting in PTI inhibition. AvrPiz-t also competes with CIPK for binding to the rice plasma membrane K^+^-channel protein AKT1, which affects K^+^ influx and suppresses ROS accumulation. OsAPIP12 interacts with both OsAPIP6 and AvrPiz-t to promote the expression of pathogenicity-related genes. AvrPiz-t can also interact with OsAPIP5 during the necrotrophic stage to decrease the protein levels of APIP5 and thus promote ETN. This ETN can be prevented in the presence of Piz-t, which upon recognition with AvrPiz-t, will activate ETI. The effector Avr-Pii has been found to inhibit ROS production by interacting with NADP-ME2 and enhance Pii-dependent ETI by interaction with two OsExo70 proteins from the exocyst complex (Fujisaki et al. 2015; Singh et al. 2016). Effectors HTR1 and HTR2 translocate to the rice nucleus to repress expression of immunity-related genes. The effector Iug4 downregulates the expression of the rice gene, *EIN2,* which is involved in the ethylene signaling pathway. By competing with the rice protein AHL1 for the binding of the promoter region of *EIN2,* Iug4 negatively regulates plant immune responses. Recognition of Avr-Pita by the R protein Pita leads to activation of ETI. Avr-Pita interacts with the rice cytochrome oxidase protein OsCOX11 to prevent ROS accumulation that would lead to an immune response. Likewise, the effector CDIP4 disrupts the OsDjA9 and OsDRP1E complex in the ER by competitively binding to OsDjA9, leading to an abundance of OsDRP1E in the host cell. Excessive OsDRP1E leads to aberrant mitochondrial fission, which in turn lowers ROS accumulation. *PAMPs* pathogen-associated molecular patterns, *PCW* plant cell wall, *PM* plant membrane, *BIC* biotrophic interfacial complex, *Ub* ubiquitin, *K*^+^ potassium ions, *PTI* PAMP-triggered immunity, *ETI* effector-triggered immunity, *ETN* effector-triggered necrosis, *ROS* reactive oxygen species, *N* nucleus, red circles, cytoplasmic effectors; green circles, apoplastic effectors. Created with BioRender.com

**Fig. 2:**
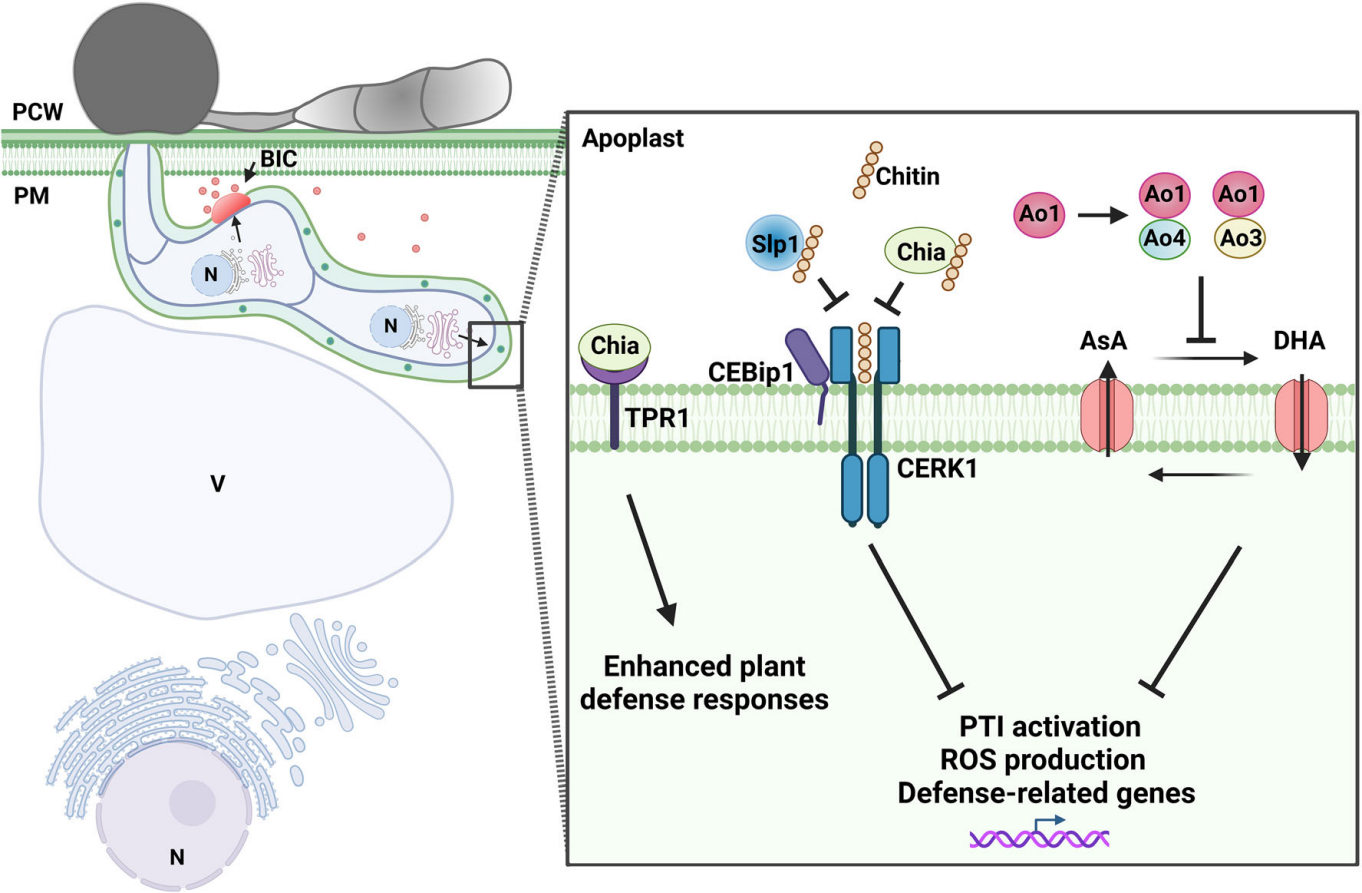
*M. oryzae* apoplastic effectors involved in evading detection by host surveillance. During early stages of *M. oryzae* infections, the rice cell surface receptor, OsCERK1 is able to recognize chitin and trigger plant immunity. Working with OsCERK1 and in the presence of chitin, OsCeBip1 can help trigger PTI and induce the expression of defense-related genes following ROS production. However, there are two *M. oryzae* apoplastic effectors, Chia1 and Slp1 that repress the activation of plant responses by intervening with CERK1-chitin recognition. The apoplastic effector, Slp1, competes for binding of chitin with OsCeBip1 in rice to prevent ROS accumulation. The effector Chia functions in a similar manner to Slp1; however, rice TPR1 proteins are able to reverse the chitin-degradati process by binding to Chia, promoting chitin accumulation, and restoring chitin-mediated plant defense responses. The apoplastic effector Ao1 disrupts the ROS levels of the cell by interfering with rice ascorbate oxidases AO3 and AO4 by decreasing the levels of dehydroascorbate (DHA). Disruption of the redox status of the apoplast leads to a decrease in ROS-triggered immunity. *PCW* plant cell wall, *PM* plant membrane, *BIC* biotrophic interfacial complex, *PTI* PAMP-triggered immunity, *ROS* reactive oxygen species, *N* nucleus, *V* vacuole, red circles, cytoplasmic effectors; green circles, apoplastic effectors. Created with BioRender.com

Currently, only a number *M. oryzae* effectors have been identified and characterized in the context of plant immunity responses. Even though some effectors remain functionally uncharacterized, some are still used as localization markers in live-cell imaging assays due to their strong localization during *M. oryzae* biotrophy growth such as Bas1 and Bas4. Here, we present a few of the most studied effectors (Figs. 1 and 2). For the *M. oryzae* effector repertoire identified thus far, review the following publications: Deb et al. (2021) and Fernandez and Orth (2018).

### The card player: ACE1

The* ACE1* (Avirulence Conferring Enzyme 1) gene encodes a polyketide synthase (PKS) and nonribosomal peptide synthetase hybrid (NRPS) (Böhnert et al. 2004). Both domains encode distinct enzymes involved in the production of secondary metabolites (Collemare et al. 2008; Fudal et al. 2007). During the early stages of *M. oryzae* infections and during host penetration, Ace1 localizes at the cytoplasm of the appressoria (Fudal et al. 2007). However, Ace1 is not secreted into the host cells, suggesting that Ace1 itself is not an actual effector. Instead, Ace1 is an enzyme that is probably involved in the biosynthesis of a molecule recognized by the plant resistant gene, *Pi33* (Böhnert et al. 2004; Fudal et al. 2007). *Pi33* recognition by *M. oryzae* strains carrying Ace1 prevents *M. oryzae* infection in rice. Interestingly, a single mutation in the β-ketoacyl synthase domain of Ace1 is enough to avoid recognition by resistant plants, suggesting that its biosynthetic activity is necessary to produce effector-like molecules (Böhnert et al. 2004). A more recent study found that the metabolite produced by Ace1 could be similar to a tyrosine-derived cytochalasan compound (Song et al. 2015). However, the identification of the specific polypeptide and the molecular mechanism involved in interacting with plant cognate R genes remain to be determined.

### The markers: Bas

The Bas (Biotrophy-associated-secreted) effectors are highly expressed during the early stages of biotrophic growth in *M. oryzae*. Bas effectors were identified a decade ago; however, where, or how Bas effectors function inside the host cell remains unknown. In addition, they do not share similarities to any other groups of known proteins. Interestingly, Bas effectors show a wide range of cytoplasmic and apoplastic localizations. For instance, the cytoplasmic Bas1 preferentially accumulates at the BIC structure, and the apoplastic Bas4 uniformly outlines growing IH (Giraldo et al. 2013; Kankanala et al. 2007; Khang et al. 2010). Despite the lack of characterization, these effectors are an excellent tool for studying protein subcellular localization *in planta*. By performing live-cell imaging of fluorescently labeled Bas1 or Bas4 effectors, new candidate effectors can be tracked by their localization in living plant cells during *M. oryzae* infection.

### The chitin lovers: Slp1 and Chia1

The *Slp1* (Secreted LysM protein 1) gene encodes a peptide of 162-amino acid residues with a secretion signal in its N-terminal. It contains two LysM domains and shares significant sequence similarities with the effector Ecp6 from *Cladosporium fulvum* and other fungal LysM proteins (De Jonge et al. 2010; Mentlak et al. 2012). Slp1 is an apoplastic effector that localizes at the plant–fungal interface, outlining the IH cells during early stages of rice blast infections (Mentlak et al. 2012) (Fig. 2). The subcellular localization pattern of Slp1 during infection is comparable to the biotrophy effector Bas4 (Mosquera et al. 2009). As *M. oryzae* moves into adjacent cells, Slp1 accumulates at the IH tips in the new invasive rice cells. A previous study using reverse genetics approaches revealed that Slp1 is required for efficient *M. oryzae* host tissue invasion. Interestingly, it was demonstrated that the Slp1 effector binds to chitin oligosaccharides to compete with the chitin elicitor receptor (OsCEBip) and acts in conjunction with the LysM receptor-like kinase (CERK1) to trigger PTI in response to chitin (Mentlak et al. 2012). The result of Slp1-chitin binding leads to the suppression of ROS production and the induction of plant defense-related genes. Slp1 encapsulates the ideal common strategy utilized by pathogens to suppress host surveillance during early invasion.

Similarly, another *M. oryzae* apoplastic effector, Chia1 (chitinase 1), has also been shown to bind to chitin in efforts to prevent chitin-triggered immunity (Yang et al. 2019) (Fig. 2). *Chia1* encodes a 397-amino acid polypeptide with an N-terminal signal peptide. Chia1 was found to be important for *M. oryzae* conidia production and germination. Additionally, through its chitinase activity, Chia1 is able to degrade chitin in the apoplast to prevent chitin-triggered immune responses. However, the rice OsTPR1 (tetratricopeptide-repeat 1) protein was also shown to competitively bind to Chia1, thereby preventing its chitinase activity from reducing the levels of chitin and promoting an immune response. Interestingly, Chia1 can also trigger surface receptors and increase ROS accumulation in the apoplast, thus promoting immune responses by acting as a PAMP.

### The promiscuous one: AvrPiz-t

The avirulent *AvrPiz-t* has an N-terminal secretion signal, encodes a predicted 108-amino acid polypeptide, and has no sequence similarity to any known proteins (Park et al. 2012). This effector is actively delivered into the BIC site and is translocated into rice cells, defining its cytoplasmic localization (Park et al. 2012). Moreover, remarkable findings demonstrate that the effector AvrPiz-t functions to suppress PTI responses and enhances plant susceptibility to *M. oryzae*. For instance, the AvrPiz-t effector translocates into rice cytoplasm and interacts with two rice RING E3 ubiquitin ligases, OsAPIP6 and OsAPIP10 (Park et al. 2012, 2016). This interaction promotes the inhibition of E3 ligase activity and OsAPIP6/10 degradation. In return, both OsAPIP6/10 positively regulate PTI responses in *M. oryzae* by degrading the AvrPiz-t effector protein. In addition, OsAPIP6/10 negatively regulates the R protein Piz-t by mediating Piz-t degradation. In the absence of Piz-t protein in rice plants, AvrPiz-t degrades OsAPIP6/10 and suppresses PTI. Silencing of OsAPIP6/10 in rice cells expressing Piz-t promotes AvrPiz-t to degrade the E3 ligases and remove the negative regulation on Piz-t, leading to a rapid accumulation of Piz-t proteins that elicit a robust HR response and the expression of pathogenicity-related proteins (Park et al. 2016). Interestingly, the OsAPIP10 degradation event led to the accumulation of two transcription factors, OsVOZ1 and OsVOZ2, which either cause inhibition of PTI responses that enhance susceptibility to *M. oryzae* in strains lacking Piz-t*,* or trigger Piz-t-dependent ETI responses following *M. oryzae* infection (Wang et al. 2021). Moreover, OsAPIP12, which encodes a protein homologous to nucleoporin 98, was identified to interact with AvrPiz-t and OsAPIP6 and is required for pathogenicity-related gene accumulation upon infection (Tang et al. 2017). Targeting of the bZIP-type transcription factor, OsAPIP5, by AvrPiz-t during the necrotrophic phase promotes the suppression of OsAPIP5 transcriptional activity and decreases its protein accumulation triggering effector-triggered necrosis (Wang et al. 2016). However, in the presence of Piz-t, ETI is activated, leading to the prevention of host necrosis. Recently, AvrPiz-t was found to target potassium (K^+^) channels to subvert plant immunity. AvrPiz-t competes for binding with the cytoplasmic kinase OsCIPK for the rice plasma membrane K^+^-channel protein OsAKT1, which causes the suppression of K^+^ inward currents mediated by OsAKT1 (Shi et al. 2018). This interaction leads to the suppression of ROS production and enhancement of plant susceptibility to *M. oryzae*. However, it is still unknown how AvrPiz-t molecularly overpowers ROS production during infection in rice plants. These pieces of evidence demonstrate that individual effectors are able to interact with multiple host targets to play distinct roles in plant immune response.

### The first one: Avr-Pita

The avirulent *Avr-Pita* was the first effector identified in the *M. oryzae–*rice pathosystem, providing the first piece of evidence that *M. oryzae* effector proteins are delivered into plant cells (46). *Avr-Pita* encodes a putative zinc metalloprotease with an N-terminal signal peptide (Jia et al. 2000). Previous studies demonstrated that Avr-Pita interacts directly and is recognized by the Pita protein inside rice cells, thus producing an HR (Bryan et al. 2000; Jia et al. 2000; Orbach et al. 2000). Avr-Pita accumulates into the BIC before delivery into the rice cytoplasm (Khang et al. 2010). Recent findings reported that Avr-Pita interacts with a cytochrome oxidase protein OsCOX11 at the mitochondria (Han et al. 2021). Avr-Pita modifies the OsCOX11 protein to regulate mitochondrial COX activity to decrease ROS accumulation in the cells. Independent experiments demonstrated that silencing OsCOX11 led to resistance against *M. oryzae* (Han et al. 2021). However, overexpression of the OsCOX11 protein boosts rice susceptibility to *M. oryzae* due to ROS inhibition and innate immunity suppression. It is apparent that the mitochondrial ROS is important in Avr-Pita-triggered immunity. However, questions arise as to how effectors are able to migrate to the host mitochondria, and if the sequence of Avr-Pita contains any regions that are conferred with mitochondrial targeting.

### The ROS regulator: Ao1

*Ao1* consists of a 603-amino acid peptide that contains an N-terminal secretion signal and three ascorbate oxidase (AO) domains. Enzymatic assays confirmed Ao1 to have AO activity. This apoplastic effector is expressed during infection and is secreted to the EIHM. Further studies showed Ao1 to play a role in regulating the redox levels of the apoplast by interacting and interfering with the activity of rice apoplastic ascorbate oxidases, OsAO3 and OsAO4 (Hu et al. 2022). In wild-type strains, OsAO3 and OsAO4 promote ROS accumulation following infection by increasing the levels of dehydroascorbate (DHA) that promotes a reduced state in the apoplast. By forming heterodimers with OsAO3 and OsAO4, Ao1 is able to reduce ROS levels in the apoplast and impair plant immune responses to promote pathogenicity, likely due to decreasing levels of DHA.

### The trans-repressors: HTR and Iug4

The cytoplasmic effectors, *HTR1* and *HTR2* (Host Transcription Reprogramming 1 and 2), encode 198- and 110-amino acid polypeptides, respectively. HTR1 and HTR2 were identified to localize at the host nucleus and act as a transcriptional repressor for genes involved in plant immunity (Kim et al. 2020). These nuclear effectors contain a C2H2 zinc finger domain to bind to the promoter region of its target genes to repress their expression during *M. oryzae* infections. The HTR repressor function on immunity-associated genes either results in enhanced susceptibility against hemibiotrophic pathogens, such as rice blast and *Xanthomonas oryzae* pv *oryzae* (*Xoo*), or increased resistance to the necrotrophic pathogen, *Cochliobolus miyabeanus* (Kim et al. 2020). These findings suggest that HTR1 and HTR2 modulate disease susceptibility/resistance by transcriptomic changes in the host based on the lifestyle of the pathogens. Another *M. oryzae* cytoplasmic effector, *Iug4* (isolate-unique genes), was also recently shown to have gene repressor activity (Dong et al. 2015; Liu et al. 2022). *Iug4* encodes a 133-amino acid polypeptide and contains a zinc binding domain at the C-terminal. Iug4 promotes disease through negatively regulating a host gene, *OsEIN2*, involved in the ethylene (ET) signaling pathway (Liu et al. 2022). Normally, an AT-hook protein, OsAHL1, can bind to the promoter region of *OsEIN2* and positively regulate the expression of *OsEIN2,* thereby enhancing the plant’s immune response via the ET signaling pathway. Iug4 was shown to have a higher affinity to the promoter region of *OsEIN2* and negatively regulate the expression of *OsEIN2.* By hindering the ET pathway, Iug4 is then able to negatively regulate plant immune responses.

### The mitochondrial disruptor: CDIP4

CDIP4 (Cell Death-Inducing Protein 4) is an *M. oryzae* cytoplasmic effector that targets the host mitochondria to suppress host immune responses (Chen et al. 2013; Xu et al. 2020). *CDIP4* encodes a 295-amino acid polypeptide, is a glycosyl hydrolase family 61-containing protein, and contains a fungal-type cellulose-binding domain. Normally in rice, the protein complex consisting of the DnaJ chaperon protein, OsDjA9 and dynamin-related protein, OsDRP1E mediates the rate of mitochondrial fission, and in turn regulates the activation of downstream immune responses (Xu et al. 2020). If OsDjA9 is knocked out, or if OsDRP1E is overexpressed, rice mitochondria become shortened and immune responses decrease, thereby making the rice more susceptible to pathogens. When CDIP4 is secreted, the protein complex is disrupted as CDIP4 competes with OsDRP1E for binding to OsDjA9, which leads to OsDRP1E accumulation and generates less ROS accumulation and prevents an enhanced immune response. To our knowledge, this is the first *M. oryzae* effector to interact with the host in the ER while influencing the mitochondrial dynamics. However, how *M. oryzae* effector, CDIP4, is able to alter organelle function remains an intriguing question.

## Unlocking the secrets of true identity

Given that most *M. oryzae* effectors are small proteins, it is difficult to discern similarities or differences based on sequence analysis alone, especially with the abundance of variations found in effector sequences. Through visualization of the quaternary protein structure of *M. oryzae* effectors using NMR spectroscopy, de Guillen and collaborators were able to identify a subset of effectors named MAX (*Magnaporthe* Avrs and ToxB like) effectors which contained structural similarities despite differences in sequences (De Guillen et al. 2015). MAX effectors consist of a variety of Avr proteins as well as effectors that share a similar β-sandwich fold to *Pyrenophora tritici-repentis* effector, ToxB. MAX effectors mirror effector groups such as LARS effectors from *Leptosphaeria maculans,* which contain structural analogs of effectors involved in suppressing plant immunity, and RALPH effectors from powdery mildews that contain a common RNase-like fold (Bauer et al. 2021; Blondeau et al. 2015; Lazar et al. 2022; Pennington et al. 2019; Spanu 2017). The grouping of analogous protein structures provides additional foundational knowledge that allows for better characterization and classification of effectors.

In more recent times, there has been a shift in the modes utilized to identify and characterize effector proteins. With computation analyses becoming more accessible, protein assessments can be conducted rapidly with a special focus on structural conservations, a feat that can more efficiently describe variations within proteins. A genome-wide computational analysis of *M. oryzae* secreted proteins using TrRosetta expanded on the grouping of effectors based on structural modeling (Seong and Krasileva 2021). This computational study analyzed 1854 secreted *M. oryzae* proteins and clustered these proteins based on common structural folds. The sensitivity of this study was also able to expand on the MAX effector family (Seong and Krasileva 2021). Additionally, through the generated predicted structures, evolutionary trajectories and predictions on protein function can be elucidated, such as with the ADP-ribose transferase protein family. The identification of novel structurally similar families could help further predict and elucidate *M. oryzae* effector functions.

The identification of *M. oryzae* effectors, their localization, function, and interactions requires researchers to take a multidisciplinary approach to examine potential effectors, with a large portion of initial work beginning *in silico* (Ray et al. 2016; Sarkar et al. 2022). With the rise of machine learning technologies, many programs can utilize gene sequences to predict secretion signals (SignalP) or overall effector characteristics such as protein size, charge, and amino acid compositions (EffectorP) (Almagro Armenteros et al. 2019; Sperschneider et al. 2015, 2016, 2018). Many programs rely on comparisons between known motifs such as RXLR motifs from oomycetes effectors, or through similarities to other effectors like MAX effectors to identify potential effector proteins (De Guillen et al. 2015; Whisson et al. 2007). Protein predictions can then be cross referenced with gene expression times that correspond to the biotrophic growth of *M. oryzae* for further validation (Sperschneider et al. 2015). Once validated, the production and purification of recombinant proteins allows for in vitro enzymatic assays to be performed to confirm the activity of these effectors based on their predicted domains (Lopez et al. 2019; Misas Villamil et al. 2019; Zhang et al. 2016). Targeted mutations can be done on the predicted active sites of the protein to further substantiate the potential enzymatic activity of *M. oryzae* effectors (Lopez et al. 2019).

Genetic manipulations of *M. oryzae* can then be performed to generate mutation strains of potential effectors which would follow the loss-of-function approach that would allow for comparison of phenotypic differences during infection (Mentlak et al. 2012). Additionally, effectors identified through computational means can be investigated in downstream applications to examine their role and function within *M. oryzae* infections *in planta*. Through fusing potential effectors with a fluorescent protein such as GFP, their localization can be elucidated and provide additional information on what is being modulated by these effectors (Khang et al. 2010). In addition, an alternative approach to visualize effectors and further characterize them is with the use of a heterologous system, such as *Nicotiana benthamiana.* However, overexpression of effectors in a heterologous system may lead to differences in the natural subcellular localization and host targets. Effector–host target candidates can also be identified using proximity labeling (PL) assays which study protein localization and interactions in living cells (Cho et al. 2020; Mair et al. 2019). These candidates can be enriched using immunoprecipitation assays and identified using mass spectrometry. This PL approach can provide a better avenue to detect low abundant and low affinity protein that are often missed using traditional methods. However, it is important to ensure that the localization of the effector is not disrupted when fused to PL enzyme while also maintaining maximal PL enzymatic activity in the studied cell. Additionally, contamination may yield false positives. Effector–host protein interactions can be supported with bimolecular fluorescence complementation, yeast two-hybrid, or using *N. benthamiana* expanding on the ways that effectors interact with the host plant (Jensen et al. 2021; Kodama and Hu 2012).

A rising method for identifying effectors is based on tracking gene expression changes throughout the early stages of *M. oryzae* infection. Specifically, Bas effectors were identified via a transcriptomic analysis (Mosquera et al. 2009). Most recently, a similar transcriptomics-based approach holistically characterized potential *M. oryzae* effectors and their expression throughout infection (Yan et al. 2023). Named MEPs (*Magnaporthe* effector proteins), this study further classified MEPs into clusters (1–10) based on the relative time of differential expression during infection. Additionally, using AlphaFold and ChimeraX, structural protein models were generated and MEPs that shared some structural similarities to the models were summed up based on their cluster number. Within the ten clusters, MEPs that contained chitin recognition protein domains, cytochrome P450 domains, and numerous glycosyl hydrolase family domains, among others, were identified (Yan et al. 2023). This comprehensive analysis of MEPs continues to depict the multifunctionality of effector proteins in *M. oryzae*. While all MEPs are expressed throughout biotrophic growth, the structural divergence of effector proteins illustrates the need to further explore both expression levels and structural complexities to begin to better dissect the intricate role of *M. oryzae* effectors during infection. Additionally, recent work characterizing the importance of codon usage for effector secretion in *M. oryzae* stresses the importance of further dissecting the function of gene expression changes at the translational level to better understand *M. oryzae* effector delivery machinery (Li et al. 2022). Future directions for delineating *M. oryzae* effectors would strive to employ more modern, streamlined methodologies that specifically incorporate both gene expressional changes and structural differences of potential effectors.

## Climate change impact on the future of rice blast

Temperature, humidity, water levels, and CO_2_ concentrations can all be impacted by climate change, and in turn, these environmental factors can influence plant–pathogen interactions. As the global climate continues to fluctuate, it is important to identify the ways in which *M. oryzae* infection may impact future global food production. While some work has been done characterizing the development of *M. oryzae* during higher temperatures, such as that increased temperatures detrimentally affected appressoria formation, not much is known about the overall effects regarding rice blast proliferation (Rajput et al. 2017). Currently, there exists a gap in understanding how global warming changes will influence *M. oryzae* infection in rice, both in the context of rice susceptibility and *M. oryzae* infection mechanisms.

In the case of rice, a study analyzing gene expression levels following infections at variable temperatures showed that rice became more susceptible to *M. oryzae*, likely due to the decreased expression of temperature-dependent resistance gene, *Pi54,* and transcription factor, *WRKY45* (Madhusudhan et al. 2019). Previously, a transcriptomics study examined the role of varying temperatures and its effects on rice infections by the bacterial pathogen *Xoo* (Sahu et al. 2020). This study identified several WRKY and ERF families of transcription factors that were differentially expressed during temperature-induced stress. Interestingly, following *Xoo* infections during higher temperatures, rice genes responsible for chromatin assembly, nucleosome organization, and chloroplast development were upregulated, suggesting that rice favors overall growth in response to elevated temperatures. Contrastingly, a recent study comparing *M. oryzae* infection in rice during normal rice growth temperatures (28 °C) and warm temperatures (22 °C) reported that while *M. oryzae* growth was more prominent at 28 °C, rice was more susceptible to *M. oryzae* at 22 °C due to decreased jasmonic acid biosynthesis that would help trigger basal immunity in rice (Qiu et al. 2022b). Exploring the ways that temperature affects rice immune responses against pathogens, especially in the scope of temperature-sensitive signaling molecules, would provide a better understanding of how *M. oryzae* may take advantage of potential rice susceptibility in the future.

In addition to temperature changes, a study that treated plants with increased CO_2_ levels before rice blast infection saw an increase in rice susceptibility (Gória et al. 2013). Recently it was found that increased humidity promoted *M. oryzae* infection in rice through a reduction in ET accumulation levels that would otherwise activate ET signaling pathways to promote rice immunity (Qiu et al. 2022a). A plant–pathogen interaction study examining the effects of elevated temperatures on *Arabidopsis* infection by *Pseudomonas syringae* pv. *tomato* (*Pst*) DC3000 depicts how salicylic acid defense responses in *Arabidopsis* are hindered through higher temperatures and promotes increased translocation of bacterial effectors into the host (Huot et al. 2017). In *M. oryzae,* a transcriptomics study looking at drought effects during rice blast disease reported that in rice that had undergone drought conditions, the expression of genes coding for small, secreted proteins in *M. oryzae* were downregulated, while genes coding for cell wall perturbing enzymes were elevated, suggesting that *M. oryzae* secretion of effectors is in some way dependent on the health of the plant itself (Bidzinski et al. 2016). While *Pst* effectors were shown to benefit from increased temperatures, more work characterizing the effects on *M. oryzae* need to be conducted to see how effector secretion and translocation are affected through the various effects of climate change.

Potential measures to enhance rice production through increasing drought conditions brought on by climate change include developing gene editing techniques to reduce the abundance of stomata in rice. Preliminary studies show that a decreased number of stomata promotes rice growth in drought conditions (Caine et al. 2019). Recently, a machine learning based prediction model was developed to forecast rice blast disease based on factors such as soil temperature, air temperature, and humidity (Liu et al. 2021). The development of mitigation strategies would benefit from additional studies conducted looking at the effects of climate change to better predict and control rice blast epidemics in the future. While gene editing can lead to the production of transgenic rice that are less susceptible to global warming changes, and computer prediction tools provide tentative models for rice blast proliferation, continued research should be conducted to dissect and discern how environmental changes driven by climate change will affect *M. oryzae*-rice interactions and how this will impact the trajectory of rice blast disease.

## Future perspectives

Deployment of effectors into host cells is a crucial part of the *Magnaporthe* infection process. Effectors influence a vast array of mechanisms and responses in the host, such as transcriptional regulation or suppression of defense triggers. As such, there is a paramount need to further characterize these effectors in efforts to better understand the modulation of host cells that occurs during *M. oryzae* infection. Blast effectors display a high level of diversity in their sequence due to the co-evolutionary arms race between the pathogen and host. Over the last few years, significant advancements have been made in cataloging new secreted effectors in *M. oryzae*. For instance, computational structural predictions and machine learning approaches have empowered a more precise annotation of fungal effectors. However, to keep up with the identification, novel high-throughput techniques are needed to deeply authenticate and characterize the new candidate effectors. Despite the progress thus far, there are still many questions about *M. oryzae* effectors that remain to be answered. Among these questions, a few major intriguing mysteries are the delivery mechanisms of effectors inside host cells and how effectors modulate host transport cargo. Once inside the host cell, what roles do effectors play, and what are the major host pathways that are affiliated with effectors? Why is the redundancy so high among *M. oryzae* effectors? Do they share similar interactors or perhaps are they needed at different infection stages to either target the same host protein or different ones to avoid recognition as infection progresses? Beyond effector proteins, non-proteinaceous entities, such as small RNA and non-coding RNA, have proven to be crucial modulators of host innate immunity. This field brings a new perspective on the effector methodology given that molecules such as small RNAs are not easily detectable by the plant surveillance mechanism, and this represents a powerful strategy by pathogens to bypass the host defense system. If *M. oryzae* uses vesicles to translocate effectors into host cells, what other molecules could they perhaps carry and deliver? Answers to these questions can bring new insights into infection mechanisms and will certainly benefit other pathosystem studies to enable the design of new mitigation approaches, perhaps through generating disease resistant lines that are more prone to activating ETI. Dissecting the avenues on how *M. oryzae* effectors promote disease gives way for potentially developing genetically engineered rice lines using CRISPR–Cas9 genome editing. Rice lines lacking effector targets not susceptible to effector modulation or containing specialized R genes that recognize effectors, thus promoting plant resistance by rendering effectors futile could be engineered. Additionally, investigations into the effects of climate change on *M. oryzae* pathogenicity and adaptability as it relates to effector expression, function and secretion are necessary to begin to comprehend how *M. oryzae* infections may shift and its potential ramifications on rice production in the future.

## Data Availability

Data sharing not applicable to this article as no datasets were generated or analysed during the current study.
